# Inflammation-Related LncRNAs Signature for Prognosis and Immune Response Evaluation in Uterine Corpus Endometrial Carcinoma

**DOI:** 10.3389/fonc.2022.923641

**Published:** 2022-06-02

**Authors:** Hongmei Gu, Jiahang Song, Yizhang Chen, Yichun Wang, Xiaofang Tan, Hongyu Zhao

**Affiliations:** ^1^ Department of Radiotherapy Oncology, Affiliated Hospital of Nantong University, Nantong, China; ^2^ Department of Radiation Oncology, The First Affiliated Hospital of Nanjing Medical University, Nanjing, China; ^3^ Department of Oncology, The First Affiliated Hospital of Nanjing Medical University, Nanjing, China; ^4^ Department of Urology, The First Affiliated Hospital of Nanjing Medical University, Nanjing, China; ^5^ Affiliated Maternity and Child Health Care Hospital of Nantong University, Nantong, China

**Keywords:** UCEC, inflammation, tumor microenvironment, prognostic signature, immunotherapy, TCGA

## Abstract

**Backgrounds:**

Uterine corpus endometrial carcinoma (UCEC) is one of the greatest threats on the female reproductive system. The aim of this study is to explore the inflammation-related LncRNA (IRLs) signature predicting the clinical outcomes and response of UCEC patients to immunotherapy and chemotherapy.

**Methods:**

Consensus clustering analysis was employed to determine inflammation-related subtype. Cox regression methods were used to unearth potential prognostic IRLs and set up a risk model. The prognostic value of the prognostic model was calculated by the Kaplan-Meier method, receiver operating characteristic (ROC) curves, and univariate and multivariate analyses. Differential abundance of immune cell infiltration, expression levels of immunomodulators, the status of tumor mutation burden (TMB), the response to immune checkpoint inhibitors (ICIs), drug sensitivity, and functional enrichment in different risk groups were also explored. Finally, we used quantitative real-time PCR (qRT-PCR) to confirm the expression patterns of model IRLs in clinical specimens.

**Results:**

All UCEC cases were divided into two clusters (C1 = 454) and (C2 = 57) which had significant differences in prognosis and immune status. Five hub IRLs were selected to develop an IRL prognostic signature (IRLPS) which had value in forecasting the clinical outcome of UCEC patients. Biological processes related to tumor and immune response were screened. Function enrichment algorithm showed tumor signaling pathways (ERBB signaling, TGF-β signaling, and Wnt signaling) were remarkably activated in high-risk group scores. In addition, the high-risk group had a higher infiltration level of M2 macrophages and lower TMB value, suggesting patients with high risk were prone to a immunosuppressive status. Furthermore, we determined several potential molecular drugs for UCEC.

**Conclusion:**

We successfully identified a novel molecular subtype and inflammation-related prognostic model for UCEC. Our constructed risk signature can be employed to assess the survival of UCEC patients and offer a valuable reference for clinical treatment regimens.

## Introduction

UCEC ranks the fourth most common in cancer incidence among females around the world (1), with unfavorable cure rate and high mortality (2). UCEC patients diagnosed in later stage have a significantly higher rate of recurrence and complications, leading to a bleak prognosis (3, 4). Traditional surgical resection, chemotherapy, and radiotherapy have been developed and have undergone constant evolution, but the overall survival (OS) has seen no significant improvement. Therefore, gaining new insight into the tumorigenesis process, pathological nature, and therapeutical agent of UCEC is vital in fighting this deadly disease.

Inflammation has a predominant effect on the immune system, creating a microenvironment conducive to cellular transformation and the spread of invasive diseases (5, 6). Research evidence shows that inflammation can also affect the occurrence and progression of cancer, *via* various pathways like oxidative stress, interleukin secretion, and pro-inflammatory transcription factors (7). Cumulative evidence also suggests epigenetics modifications like DNA methylation, histone modification, remodeling of chromatin, and regulation *via* non-coding RNAs can modulate the balance of inflammation and accelerate the tumorigenesis process (8). LncRNAs are a novel kind of RNA that can regulate cellular signaling pathways in UCEC (9). For example, LncRNA NEAT1 is found to drive endometrial cancer progression by targeting the oncogene STAT3 (10). Similarly, Wang et al. reported another LncRNA NR2F1-AS1 is able to assist miR-363 to target SOX4, thus increasing the risk of endometrial cancer (11). Considerable research has shown the role of inflammatory pathways in cancer is regulated by a number of lncRNAs. Hu et al. disclosed that upregulation of lncRNA XLOC-000647 can inhibit the expression of NLRP3 inflammatory vesicles, which in turn suppresses the metastasis of pancreatic cancer cells (12). In breast cancer, lncRNA NKILA was proven to interact directly with NF-κB to mediate inflammatory pathways and thereby inhibit tumor metastasis (13). 

The TME supports the intricate process of tumorigenesis by modulating various functionally interlinked cells and non-cellular components (14, 15). Numerous previous studies have reported modulation function of immune and inflammatory cells on UCEC cells. These factors woven together provide a welcoming host for the UCEC cells, and greatly enhance their capability to replicate, invade, and resist drugs. Previous research has discovered numerous potential hallmark signals and proteins. For example, the overexpression of CXCL12/CXCR4 is reported to be correlated with unfavorable prognosis in UCEC patients (16). Utilizing the ESTIMATE and CIBERSORT algorithms, Xu et al. discovered that cell-cell chemokine receptor 2 (CCR2) can facilitate the recruitment of monocytes and macrophages into the TME, affecting the prognosis of UCEC patients. On the other hand, MSI status implies the existence of high-level TIL-infiltration, taking mismatch repair defect into consideration. Prognostic effects of biomarkers varies on molecular subtypes: In p53-mutant UCEC, Treg is an independent prognostic factor, while in NSMP, WHO-grading has unreplaceable prognostic value (17). Cumulating evidence implies additional factors are needed to drive the tumorigenesis process apart from merely genetic mutations, and the microenvironment-derived factors may be exactly the missing puzzle piece. However, the precise mechanism and molecular signal remains disputed, and calls for additional research. Any new insight into the nature of TME can potentially improve the precision of prognosis prediction or reveal promising therapeutic targets.

In this project, we determined a novel molecular subtype and a risk signature based on IRLs which were tightly correlated with survival outcome of UCEC cases. Moreover, our proposed risk model can reflect the immune status and evaluate the benefits of immunotherapy and chemotherapy.

## Methods

### Data Acquisition

Transcriptome and RNA-seq data of UCEC patients were retrieved from the TCGA database (https://portal.gdc.cancer.gov/) and the transcriptome data files were in “FPKM” format. Five hundred eleven UCEC patients with clinicopathological information were used for analysis. The exclusion criteria were set as follows (1): histologic diagnosis is not UCEC (2); samples without completed clinical data; and (3) survival time of less than 30 days. In total, 511 UCEC patients were randomly divided equally into the training cohort (256 patients) and a validation cohort (255 patients) by utilizing the caret R package. Detailed annotation of the tumor samples complete with clinical and pathological information can be found in **Table S1** (P > 0.05, Chi squared test).

### Determination of the IRLs

The list of 200 inflammation-related genes was acquired from the GSEA database (http://www.gsea-msigdb.org). We screened the IRLs by Pearson’s correlation analysis, and 636 IRLs were identified. The process applied the criteria of |Pearson R| >0.5 and p <0.001.

### Gene Set Enrichment Analysis

The training set was applied to establish the IRLPS, and validation of aforementioned model is made using the testing set and entire set. Completed with survival data retrieved from TCGA, we explored the prognosis value of IRLs, and univariate Cox regression was used to filter 27 prognostic IRLs. The next step was to understand the biological processes that these IRLs are involved in. The “ConsensusClusterPlus” package was used to divide UCEC patients into groups based on clinical outcome and pathological classifications (18). Then, gene set enrichment analysis (GSEA) was used to determine which process or pathway made a difference in the outcome (19, 20). KEGG can identify predefined gene sets activated or deactivated, p-value were determined by performing 5000 permutations according to the gene set. A pathway with a p-value < 0.05 was considered as significant.

### Estimating of Tumor-Infiltrating Immune Cells

CIBERSORT was employed to calculate the abundance of 22 types of immune-related tumor-infiltrating cells in all samples (21). The proportion of data generated will be used for further analysis. ESTIMATE algorithm was used to screen each sample, computing the proportion of immune and stromal components in the TME (22); the immune score and stromal score are results of these algorithms. The ESTIMATE score is determined by combining immune score and stromal score. The value of these scores has a positive connection with the proportion of stromal, immune, and the sum of the first two, respectively. With the “GSVA” package in R, we calculated the abundance of 16 immune cells in the microenvironment, represented by the infiltration scores, and the activities of 13 immune-related pathways between the high-risk and low-risk groups *via* single-sample gene set enrichment analysis (ssGSEA) (23).

### Establishment of the IRLPS

To build our risk model, we chose the LASSO Cox regression to generate the optimal choice of coefficients and variants that constitutes the risk score equation (24). A 10-fold cross validation with minimum criteria was applied to optimize the signature. The remaining non-zero features were utilized to build the final model. LASSO regression was conducted with the R package “glmnet” (25). Generated from LASSO, these coefficients made up our risk score equation:


$$risk\;score=\sum_{i=1}^{n}coefficient_{i}*\;expression\;levelof\;IRL_{i}$$

### Acquisition of Clinical Specimens

The 32 specimens (16 tumor samples and 16 normal samples) used for quantitative PCR assay were acquired from 16 consenting patients at Maternity and Child Health Care Hospital of Nantong University. Our research protocol was approved by the Ethics Committee for Clinical Research of the Maternity and Child Health Care Hospital of Nantong University. All research was conductedin strict adherence to the Declaration of Helsinki.

### RNA Extraction and Quantitative Real-Time Quantitative PCR (RT-qPCR) Analysis

The total RNA was extracted from the aforementioned 32 samples using TRIzol reagent (Thermo Fisher Scientific, Waltham, MA, USA), and then evaluated for RNA structure integrity using the Agilent Bioanalyzer 2100 (Agilent Technologies, Santa Clara, CA, USA) with the RNA 6000 Nano Kit. By utilizing the High-Capacity cDNA Reverse Transcription Kit (Thermo Fisher Scientific), we therefore synthesized complementary single-stranded DNA and then performed the real-time quantitative analysis by the SYBR Green PCR Kit (Thermo Fisher Scientific). The relative transcription level was assessed with the 2^-△△Ct^ method, Ct represents the cycle threshold of each IRL. All programs and procedures were conducted on the basis of the instructions offered by the manufacturer. Primer sequences that were used can be found in **Table S2**.

### Validation of the IRLPS

Now that we have this risk score to forecast the OS of UCEC patients, the next step was validation of our model. Again, our patients were assigned to groups assigned by the median risk score, and then we checked whether there was a statistical difference in OS between groups. The accuracy of IRLPS was presented in the form of receiver operating characteristic (ROC) curve and the area under the ROC curve (AUC value), generated by using R package “survivalROC” (26). The model was then validated with the testing data set and entire set, its effectiveness was measured by ROC curve, compared with clinical or pathological criteria alone and combined as a risk indicator. The Kaplan-Meier analysis was performed with the “survival” package (27). The risk curve and scatter plot were generated to illustrate the risk score and survival status of each sample. The heatmap indicated the expression pro-files of the signature in the two groups. Principal components analysis (PCA) was applied to dimensionality reduction (28). In order to identify independence of IRLPS, we employed both univariate and multivariate Cox regression analyses. To further verify the prediction power of our risk score, we performed stratified analysis by clinical classifications. We built the nomogram on the basis of the outcomes of multivariate Cox regression to predicting 1-, 3-, and 5-year survival probability through “rms” package (29). The calibration figures represent the consistency of our prediction with reality.

### Mutation Analysis

Patient characteristics and their sequencing status were retrieved from TCGA. The fall plots give visual hints of the 20 most frequent mutated genes, made by the R package “maftools” (30). Additionally, the stemness of tumor cells in each endometrial carcinoma sample wasdetermined by one-class logistic regression, represented by the stemlike indices (31).

### Immunophenoscore Analysis

Derived by z-scores of iconic genes related to immunogenicity, immunophenoscore (IPS) is a representation of a sample’s overall immunogenicity. The IPSs of UCEC patients were extracted from the Cancer Immunome Atlas (TCIA) (32) (https://tcia.at/home). Based on four main classes of genes (PD1, PD-L1, PD-L2, CTLA-4), MSI was generated by machine learning in an unbiased manner. Together, IPS and MSI give an overview of the immunophenotype.

### Chemotherapy Response and Drug Sensitivity Analysis

The response of UCEC patients to therapeutic agents, whether chemotherapy or small molecular agents, were found in a public database called Genomics of Drug Sensitivity in Cancer (GDSC; https://www.cancerrxgene.org). The half-maximal inhibitory concentration (IC50) was taken as an index to measure the sensitivity (33). Up to 60 different cancer cell lines that originated from nine different cancers were made available as per request *via* the CellMiner interface (https://discover.nci.nih.gov/cellminer) (34, 35). Correlation between the expression of previously mentioned genes with prognostic value and drug sensitivity were explored using Pearson correlation analysis.

### Statistical Analysis

All the analyses were processed using R software (version 4.1.0) (36). Student’s t-test was applied to perform the group comparisons between subgroups separately. To uncover potentially significant differences in OS between risk score defined groups, Kaplan-Meier analysis and log-rank tests were used. The correlation of the risk score generated by our model with stemness score, stromal score, immune score, and drug sensitivity was tested by Spearman or Pearson correlation analysis. a p-value < 0.05 is considered significant.

## Results

### Data Acquisition and Generation of Differential Expressed lncRNAs

**Figure 1:**
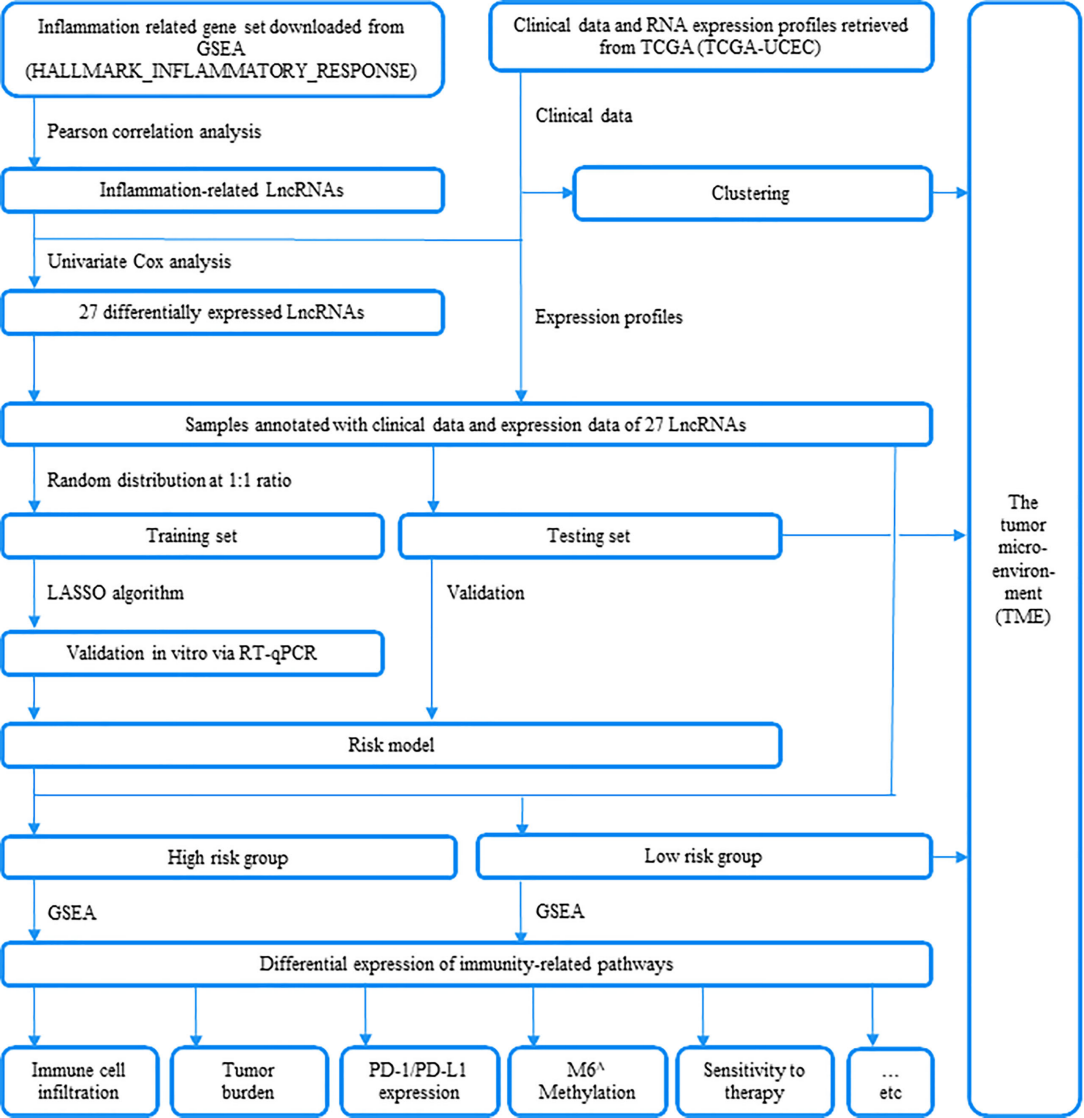
An outline of this research is depicted in this plot.

**Figure 2:**
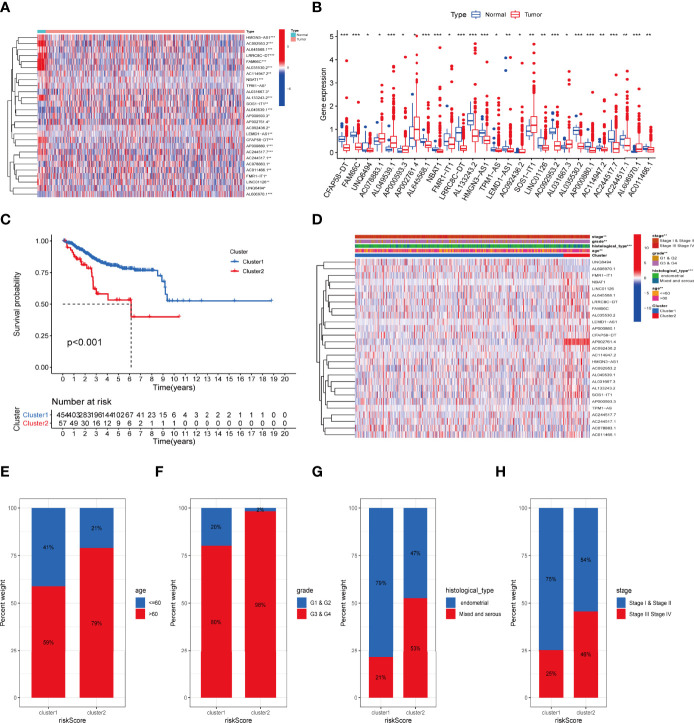
The association between the transcription level of IRLs and clinicopathological and prognostic features of the UCEC patients. **(A, B)** The transcription levels of 27 differentially expressed IRLs between the tumor and normal samples were visualized by heatmap and boxplot. **(C)** The overall survival of UCEC patients in the two clusters was calculated by Kaplan-Meier curves. **(D)** The transcription levels of 27 differentially expressed IRLs between the two clusters with clinical features were shown in heatmap. **(E-H)** The ratio of different age **(E)**, grade **(F)**, histological type **(G)**, and stage **(H)** in the groups. *P < 0.05, **P < 0.01, ***P < 0.001.

The total workflow of this research is shown in **Figure 1**. In short, we first retrieved transcriptome and clinical data of the UCEC patients from the TCGA database, and inflammation-related gene sets from the GSEA database. Combining them, we subsequently used Pearson correlation analysis to screen 636 lncRNAs to find those most closely correlated with prognosis. Twenty-seven lncRNAs were identified as prognostic *via* univariate Cox regression (**Table S3**). The expression levels of 27 IRLs in UCEC and normal tissues were evident (**Figures 2A, B**).

### Identification of Inflammation Molecular Subtype in UCEC

To determine the inflammation-associated subtype, all UCEC cases were subject to consensus clustering method based on 27 IRLs. **Figures S1A, S1B** show the respective cumulative distribution function (CDF) of consensus clusters ranging from k = 2 to 9 and the corresponding area under curve. As is shown, k = 2 is the choice to divide the UCEC patient in order to reach maximum consensus within clusters (**Figures S1A, B**). Tracking plots for k = 2 to k = 10 is exhibited in **Figure S1C**, and relative change in area under CDFG curve is demonstrated in **Figure S1D**. According to the expression levels of the 27 IRLs, 511 UCEC patients were clustered into cluster 1 (n1 = 454) and cluster 2 (n2 = 57). As suggested by **Figure 2C**, patients in cluster2 presented a dismal outcome compared to those in cluster 1 (p < 0.001). We then assessed the correlation between clusters and clinical parameters of UCEC patients. (**Figures 2D–H**).

### Immune Activity Analysis of Molecular Subtype

**Figure 3:**
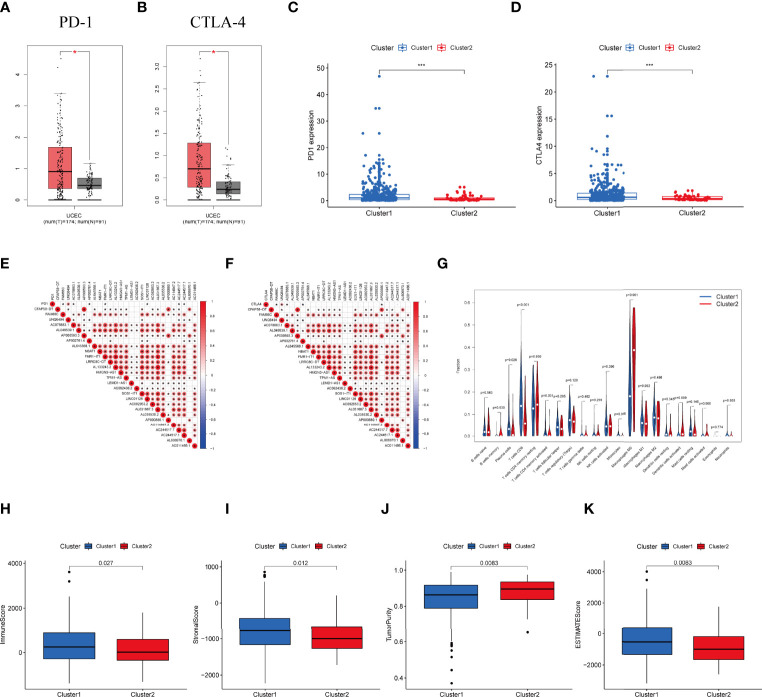
Differential expression profile of immune checkpoint related genes and TME components between clusters. **(A)** The expression of PD-1 in normal and UCEC tissues. **(B)** The expression of CTLA-4 in normal and UCEC samples. **(C)** The expressionion level of PD-1 in the clusters. **(D)** The expression level of CTLA-4 in the clusters. **(E)** The correlation of the transcription levels of IRLs and PD-L1, red circle means positive relationship. **(F)** The correlation of the transcription levels of IRLs and CTLA-4, red circle means positive correlation. **(G)** The infiltrating levels of 21 immune cell types in two clusters. **(H–K)** The **(H)** Immunescore, **(I)** Stromalscore, **(J)** Tumor purity score, and **(K)** ESTIMATEscore in cluster 1 and cluster 2. *P < 0.05, ***P < 0.001.

Expression of PD-1 and CTLA-4 were compared between tumor and normal tissue samples in UCEC patients. Our result revealed that the expression of PD-1 and CTLA-4 in UCEC tissues was upregulated (P < 0.001, **Figures 3A, B**) compared to their normal counterparts. In regard to the consensus clusters, we observed the higher expression of PD-1 and CTLA-4 in cluster 1 (**Figures 3C, D**). In addition, the expression of two immune checkpoints was positively related to the expression levels of FAM66C, UNQ6494, AC078883.1, AP002761.4, FMR1-IT1, LINC01126, AC244517.7, and AC244517.1 (**Figures 3E, F**). The differential infiltration of 22 immunocytes between the two clusters is shown in **Figure 3G**.

Moreover, we assessed the immune microenvironment value of the UCEC samples. The results suggested that cluster 1 displayed a higher immune microenvironment score, whereas cluster 2 had the higher level of tumorpurity (**Figures 3H–K**). Meanwhile, GSEA was employed to detect the TME phenotype of the two clusters. We found that immune-related pathways were mainly enriched in cluster 2. The results reveal that the top 10 pathways enriched in cluster 1, while cancer-associated pathways were activated in cluster 2 (**Figure S2A**).

### Establishment of Prognostic Signatures Based on IRL

In the training dataset, univariate Cox regression was first used to filter 27 prognostic IRLs. Then we employed LASSO algorithm to remove overfitting genes and selected five lncRNAs to create a signature (**Figure S3**), including HMGN3-AS1, LEMD1-AS1, AP000880.1, AC244517.1, and AC011466.1. The complete formula was as below: Risk score = (0.286 × HMGN3-AS1) + (0.065 × LEMD1-AS1) + (0.854 × AP000880.1) + (0.048 × AC244517.1) + (0.600 × AC011466.1). Next, the expression pattern of five hub markers between tumor and normal specimens was confirmed. Both five lncRNAs were downregulated in tumor tissues based on TCGA-UCEC dataset (**Figure S4A–E**). We further examined the expression level of five lncRNAs in clinical samples. The results indicated that only AP000880.1 and AC244517.1 showed the expression difference between two groups (**Figure S4F–J**).

Subsequently, UCEC patients were divided into high-risk and low-risk groups. PCA analysis shows satisfying separation efficacy in the training, testing group, and entire cohort (**Figures S5A–C**). Sankey diagram presented the association among cluster, risk score, and survival outcome of UCEC cases (**Figures S5D**).

**Figure 4:**
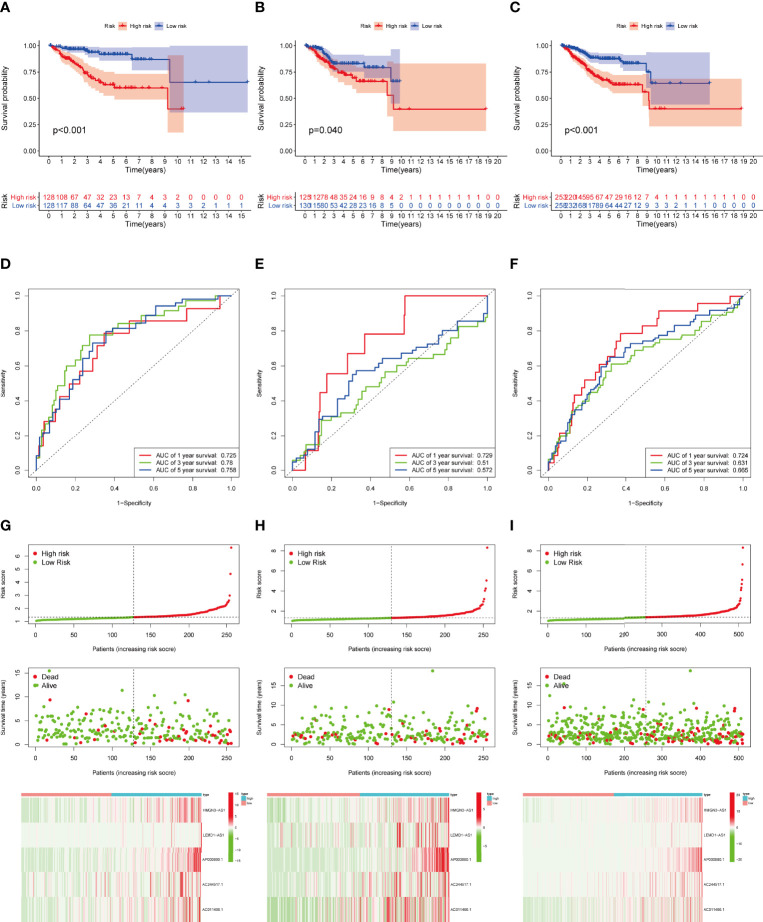
Construction and validation of IRLPS. **(A-C)** Survival analysis for patients in the **(A)** training, **(B)** testing, and **(C)** entire cohort. **(D-F)** ROC curves measuring the predictability of the signature in the **(D)** training set, **(E)** testing set, and **(F)** entire cohort. **(G-I)** Distribution of risk score, survival status, and heatmap of the transcription levels of five prognostic signatures in the **(G)** training set, **(H)** testing set, and **(I)** entire cohort.

Then we validated this model in the test set and entire set. In the training set, Kaplan-Meier curves uncovered the significant difference of prognosis between two high groups (**Figures 4A**). The AUC value of 1-, 3-, and 5-year OS were 0.725, 0.780, and 0.758, respectively (**Figure 4D**). The performance of model was shown in **Figure 4G**. At the same time, the test set and entire set were utilized to confirm our proposed signature (**Figure 4**)

**Figure 5:**
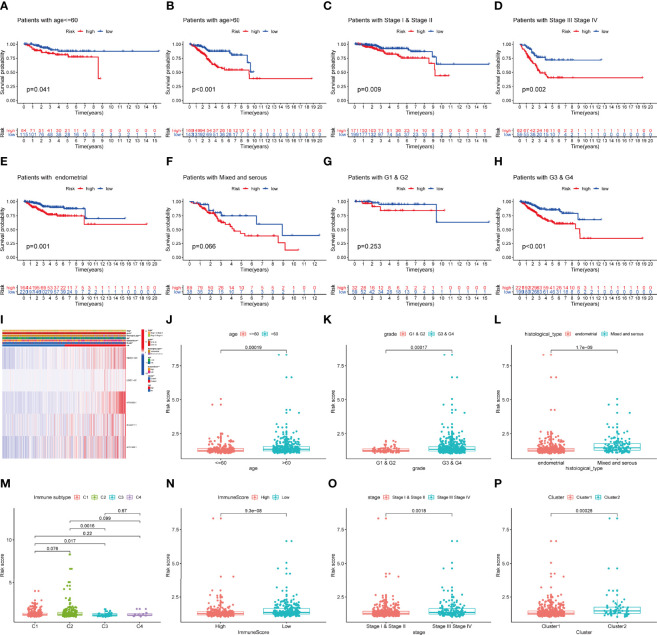
The prognostic value of IRLPS in stratified patient groups, and correlation of IRLPS with clinicopathological features and immunescore. IRLPS showed satisfactory prediction performance in patients regardless of **(A, B)** age and **(C, D)** stage, **(E, F)** histological type and **(G, H)** grade. **(I)** Heatmap and clinical features of the groups. (J–P) Distribution of IRLPS stratified by **(J)** age, **(K)** grade, **(L)** histological type, **(N)** immunological subtype, **(M)** Immunescore, **(O)** tumor stage and **(P)** cluster. *P < 0.05, **P < 0.01 ***P < 0.001.

In addition, we explored the predictive ability of the model based on subgroup analysis. **Figure 5** reveals that signature showed the favorable power in age and stage subgroups. Furthermore, we plotted a heatmap as an overview of the relationship between clinical features and risk score (**Figure 5I**). The risk score was significantly different between some clinical factors including age, grade, histological type, immune subtype, immunescore, stage, and cluster (**Figures 5J–P**).

### Development of a Prognosis Nomogram

**Figure 6:**
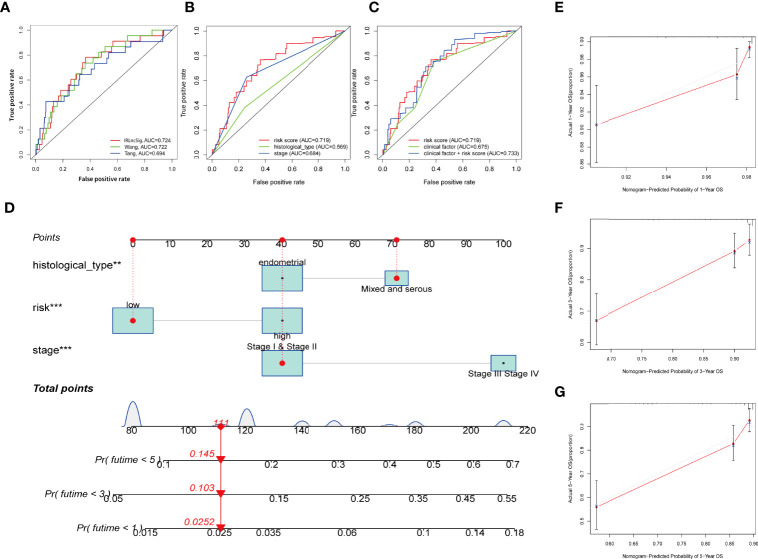
Establishing the IRLPS based on risk score and clinical factors, and validating it in calibration plot. **(A)** ROC plot indicates that IRLPS is superior in predicting the prognosis in UCEC patients than previous works. **(B)** IRLPS is also more superior in prediction accuracy than histological type or tumor stage alone. **(C)** Combining IRLPS with clinical factors is better yet. **(D)** A nomogram to illustrate the IRLPS, a risk model to predict endometrial carcinoma patient prognosis basing on aforementioned IRLPS, and clinical factors. **(E–G)** Calibration curves showing the favorable performance of nomogram. **P < 0.01 ***P < 0.001.

As uncovered by Cox regression analysis, our constructed signature was proven to be an independent factor in training, testing, and entire sets, respectively (**Table S4**). Previous work has already explored the prognostic value of lncRNAs in UCEC, and yielded promising results (37, 38). In this research, risk score based on IRLs (IRLPS) is more superior in prognostic accuracy compared to its predecessors (**Figure 6A**). Next, we conducted the univariate and multivariate methods and found that the histological type and stage are also independent prognostic factors in UCEC (**Figure 6B**). We then compared our model and clinical characteristics in pursuit of greater efficacy for predicting clinical outcome (**Figure 6C**) and observed that taking clinical factors into consideration presented higher AUC value. To further expand the forecasting ability, we established a nomogram by combining risk score and other clinical traits (**Figure 6D**). Each of them is mapped to a bar representing range of value they contribute to prognostic risk. To test the sensitivity and specificity of the nomogram, we established calibration curves, which implies there was a close fit between the prognosis and real curves (**Figures 6E–G**).

### GSEA Enrichment of Risk Model

**Figure 7:**
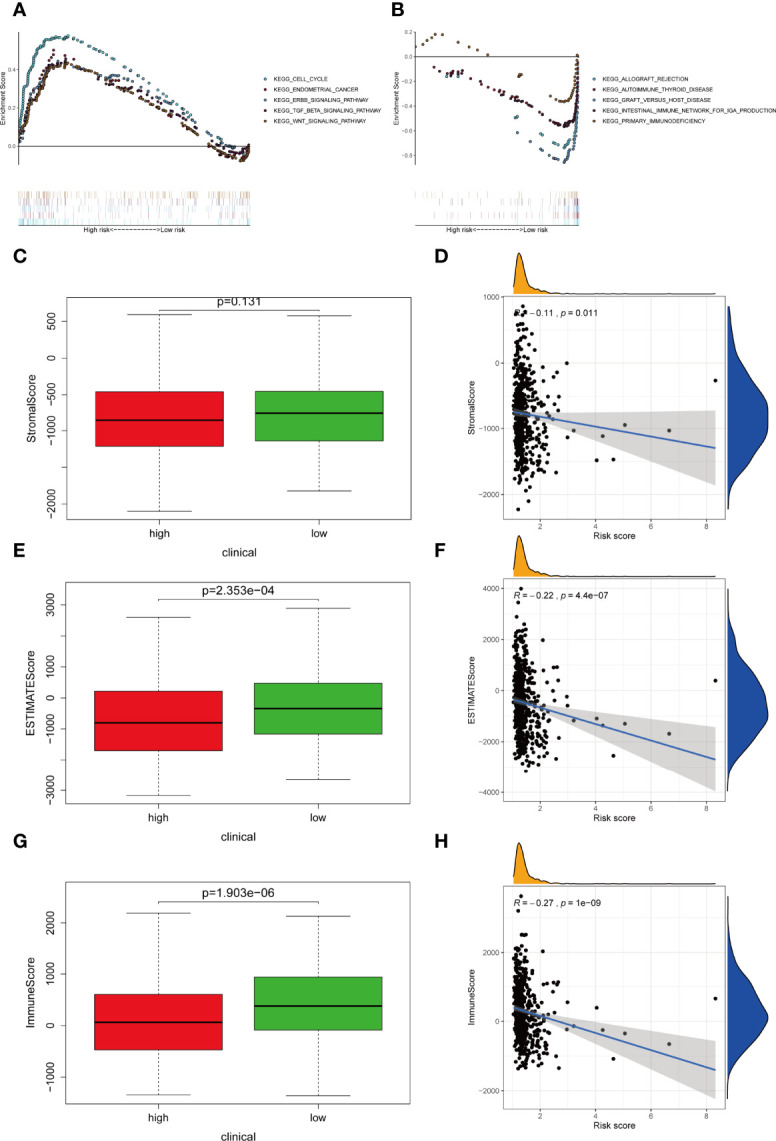
Differentially activated pathways and immune infiltration between the groups. **(A, B)** Multiple GSEA analysis was conducted to predict the potential functions and pathways involved in **(A)** high-risk and **(B)** low-risk groups. **(C, D)** Stromal score does not differ significantly between the groups. However, correlation analysis implies significant relationship between stromal score and IRLPS. **(E, F)** ESTIMATE score differs significantly between the groups, and correlation analysis implies a significant relationship between ESTIMATE score and IRLPS. **(G, H)** Immunescore differs significantly between the groups, and correlation analysis implies a significant relationship between Immunescore and IRLPS risk score.

GSEA revealed the top five active pathways in the high-risk group including cell cycle, endometrial cancer, ERBB signaling pathway, TGF-β signaling pathway, and WNT signaling pathway (**Figure 7A**), while the low-risk group included allograft rejection, autoimmune thyroid disease, graft versus host disease, intestinal immune network for IgA production, and primary immunodeficiency (**Figure 7B**).

### Immune Landscape Between Two Risk Groups

**Figure 8:**
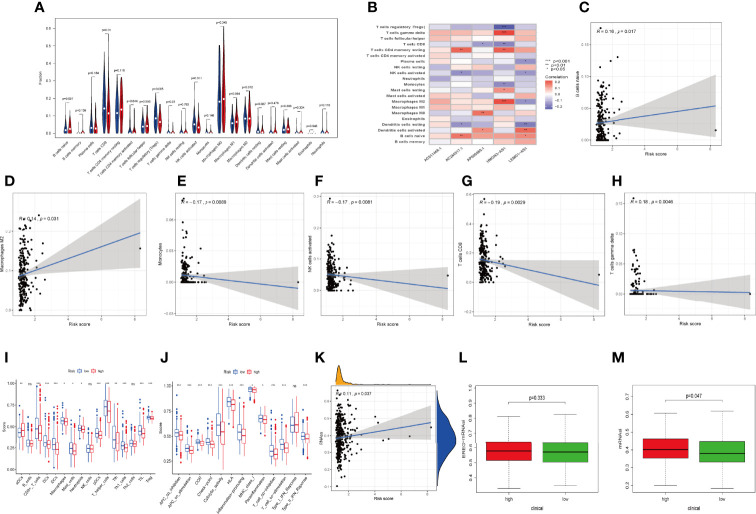
Relationships between IRLPS and different aspects in the immune microenvironment, including infiltration abundances and activation status of immune cells and cell stemness. **(A)** Violin plot depicts 21 immune cell types that is differently distributed in high and low risk IRLPS risk score groups. **(B)** The correlation of IRLs expression and infiltration abundance of immune cells, visualized by heatmap. **(C–H)** The correlation of 6 immune cell types with the 5 IRLs in our risk signature. **(I, J)** ssGSEA reveals significant difference in **(I)** immune cell abundance and **(J)** activation of immune processes between the groups. **(K)** Correlation analysis implies significant relationship in cancer cell stemness represented by methylation of RNA (RNAss) with risk score. **(L)** No significant difference in epiregulin mRNA stemlike indices (EREG mRNAsi) between the groups. **(M)** The mRNA based stemlike indices (mRNAsi) is significantly different in the groups. *P < 0.05, **P < 0.01 ***P < 0.001, ns indicates no statistical difference.

Considering that the IRLPS were associated with the immune-related pathway, we detected the immune status of two subgroups. Firstly, we noticed that the low-risk patients had a higher TME score than the high-risk patients (**Figures 7C, E, G**). Also, correlation analysis verified the above results (**Figures 7D, F, H**). Subsequently, the immune landscape of the two risk groups was mirrored by **Figure 8A**. The relationship between five model lncRNAs and immune cell infiltration was further analyzed (**Figure 8B**). Correlation method showed that the infiltration levels of B cells and Macrophages M2 were positively associated with risk score, while risk score had a negative correlation with the proportions of monocytes, activated NK cells, and CD8 T cells (**Figures 8C–H**).

ssGSEA also presented the similar immune status of all patients (**Figure 8I**). Additionally, we found that the high-risk group had lower immune activity, which might be a potential explanation for the dismal outcome of cases with high risk (**Figure 8J**). Previous reports have demonstrated that patients with poor immune activity tend to have worse prognosis (39–41).

RNA stemness score (RNAss) is an effect index representing tumor stemness (42). All three types of stemness-related indicators uncovered that the high-risk group had a higher tumor stemness (**Figures 8K–M**).

### Immunotherapy Response Analysis of IRLPS

**Figure 9:**
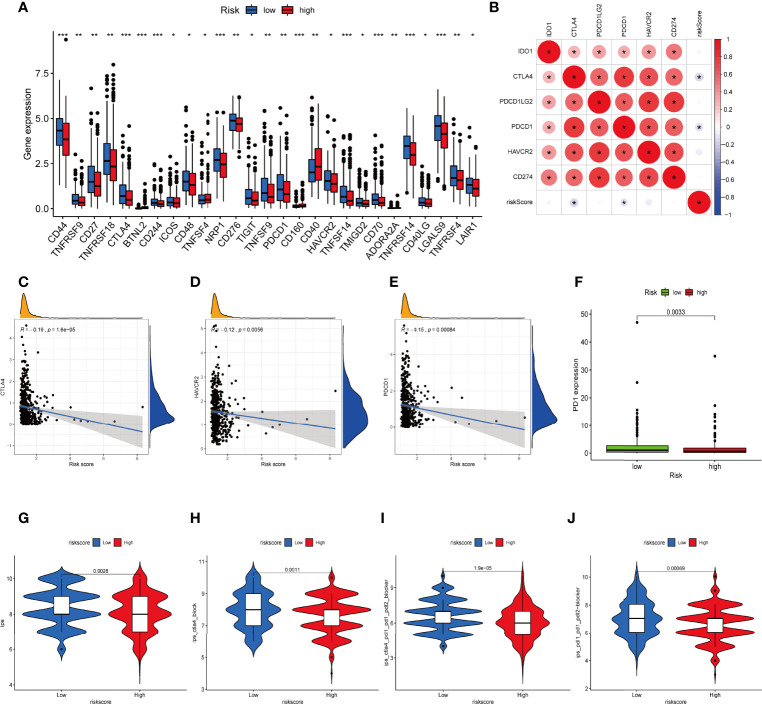
Difference in ICGs expression in the two IRLPS groups. **(A)** The boxplot shows the correlation of ICGs and risk score. **(B)** Correlation between expression of ICGs and IRLPS. **(C-E)** Correlation analysis reveals expression levels of ICGs **(C)** CTLA-4, **(D)** HAVCR-2, and **(E)** PDCD1 are negatively related to IRLPS risk score. **(F)** Boxplot illustrates significantly higher expression of ICG PD-1 in the IRLPS low-risk group than in the high-risk group. **(G–J)** IPS scoring reveals **(G)** IPS, **(H)** IPS-CTLA4, **(I)** IPS-CTLA-4/PD-L1/PD-1/PD-L2, and **(J)** IPS-PD-L1/PD-1/PD-L2 scores were all significantly higher in the low-risk group. *P < 0.05; **P < 0.01; ***P < 0.001.

Considering the crucial role of immune checkpoints in immunotherapy, we collected 27 immune checkpoint genes (ICGs), including CD44, TNFRSF9, CD27, TNFRSF18, CTLA4, CD244, ICOS, CD48, NRP1, CD276, TIGIT, TNFSF9, PDCD1, HAVCR2, TNFSF14, TMIGD2, CD70, TNFRSF14, CD40LG, LGALS9, TNFRSF4, and LAIR1. The results suggested that most immune checkpoints were highly expressed in the low-risk group (**Figure 9A**). The relationship between six classical immune checkpoints and risk score are shown in **Figure 9B**. Meanwhile, we observed that high risk score was positively correlated with the expression levels of CTLA-4, HAVCR-2, and PD1 (**Figures 9C–F**). Moreover, IPS algorithm was employed to determine the immunogenicity of the two groups. Four types of IPS-related scores were lower in the high-risk group (**Figures 9G–J**).

The comparison in the expression of m6A-related markers between the two groups indicated that the expressions of all markers were significant except for FTO, YTHDC2, and ALKBH5 (**Figure S6A**). Mismatched repair genes (MRGs) have long been established as predictors for immunotherapy benefits (43, 44). Here, we found that four MRGs (MSH2, MSH6, PMS2, and MLH1) were highly expressed in the high-risk group.

### TMB Analysis of the IRLPS

**Figure 10:**
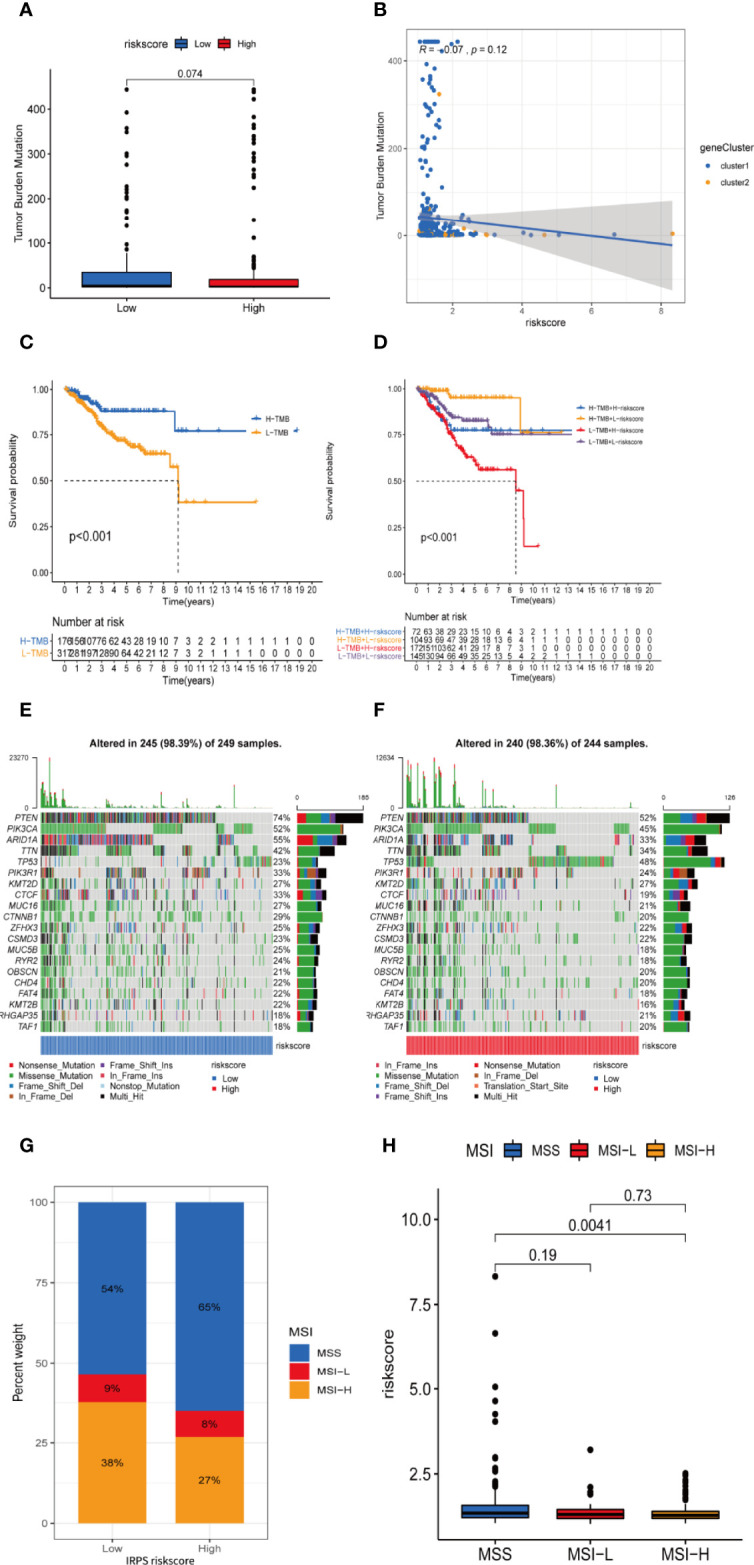
TMB and microsatellite instability are negatively correlated to IRLPS risk score, and can contribute to more significant prognostic discrimination combined with risk score. **(A)** Boxplot shows higher TMB in the low-risk group. **(B)** Correlation analysis implies TMB is potentially negatively related to IRLPS risk score. **(C)** Kaplan-Meier analysis indicates unfavorable outcome for low TMB patients. **(D)** Patients with lower TMB and higher risk score have significantly more pessimistic outcomes. **(E, F)** Mutation profile in **(E)** low and **(F)** high risk score groups. **(G)** IRLPS high-risk group has higher proportion of MSS and lower proportion of MSI-H. **(H)** Divided by microsatellite status, the MSI-H group has significantly lower risk score.

TMB level was yet another factor that can’t be ignored in predicting the response to immunotherapy. Here, we examined both subgroups and compared their TMB levels. **Figures 10A, B** showsthat the TMB was negatively related to risk score. Subsequently, the patients were assigned into unique clusters in terms of the TMB value. Survival analysis showed that the high-TMB group displayed a favorable outcome (**Figure 10C**, p < 0.001). Furthermore, we noticed that patients with low TMB as well as a high-risk score showed the worst clinical outcomes (**Figure 10D**, p < 0.001).

An overview of somatic variants provides an insight into the scatter patterns of the top 20 most frequently mutated genes. The mutational landscapes presented that the top 20 mutated genes were the same in both groups, led by PTEN, PIK3CA, and ARID1A (**Figures 10E, F**). In this case, we also evaluated the MSI of UCEC patients. As is shown in **Figure 10G**, the prevalence of high instability of microsatellites (MSI-H) was higher in the low-risk group (38% vs. 27%), while the prevalence of stable microsatellites (MSS) was higher in the high-risk group (**Figures 10G, H**). This implies a negative correlation between microsatellite instability and IRLPS risk score.

### Chemotherapy Response Analysis of IRLPS

**Figure 11:**
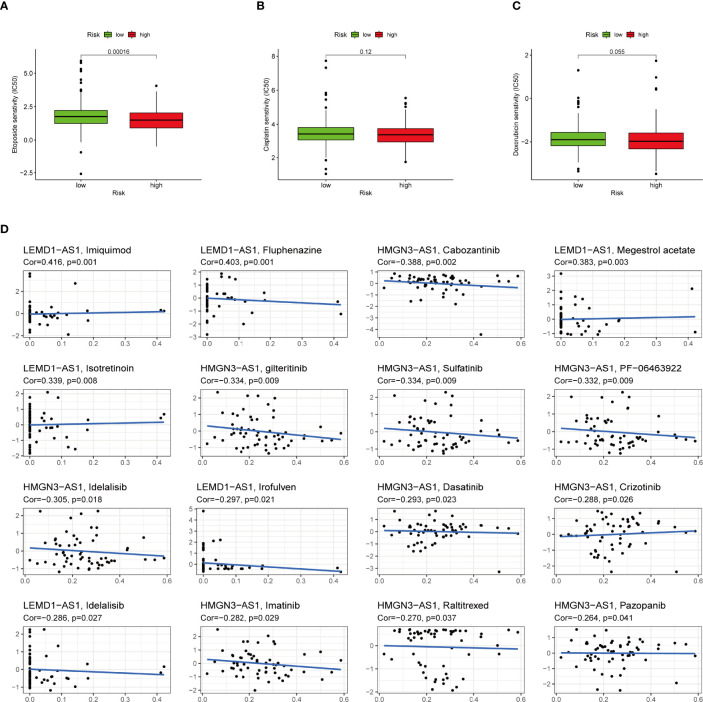
High-risk group is generally less sensitive to chemotherapy and several potential small molecule therapeutical agents targeting the IRLs. **(A-C)** Correlation of risk score clustering and chemotherapy response. Response to **(A)** etoposide, **(B)** cisplatin, and **(C)** doxorubicin is generally less significant in high-risk patients. **(D)** Several small molecular agents are found to be able to counter the expression of these IRLs.

To select potential chemotherapeutics for UCEC patients, we calculated the IC50 of three common chemotherapeutic drugs in two groups and assessed the correlation between IRLs and chemotherapeutic drugs. The results showed that etoposide and doxorubicin had higher IC50 in the low-risk group (**Figures 11A–C**). Five model lncRNAs were closely related to the sensitivity of chemotherapeutic drugs (P < 0.05) (**Figure 11D**).

## Discussion

UCEC is among the most encountered threat to the female reproductive system. As for now, the therapy in clinical use is based on the clinical staging system, which is far from satisfactory, partly because it neglects the heterogeneity of UCEC patients and interactions in the TME (45).

Numerous reports have suggested that the inflammatory chemokine ligand/receptor axis promotes UCEC proliferation, progression, and metastasis (46). The inflammation process play an indispensable role in the progression and metastasis phase (47). Sorted by TME profiling signatures, the solid tumor is classified into three types, the T cell inflamed, the “desert,” and the “excluded” phenotype (48). The context of this specified immune landscape is closely associated with response to immunotherapy. Therefore, understanding the extent to which the tumor is inflamed is of vital significance, and should be the starting point of effective immunotherapy. Currently, there is no widely recognized indicator of inflammation activity on the epigenetics level. Our work is aimed to contribute to a more comprehensive and decisive means to predict and optimize the efficacy of immunotherapy.

The risk signature constructed in our study is a reliable and robust marker to predict the survival outcome of UCEC patients. Besides this, the signature was robustly associated with immune infiltration levels, TMB scores, and chemo-sensitivity. Our research further investigates the role of LRLs in the tumor microenvironment, pharmaceutical landscape, and prognostic prediction in UCEC, providing a novel insight for future research and clinical practice.

In this research, we first determined a novel inflammation-associated subtype for UCEC. All patients were classified into two clusters which had significant differences in both prognosis and immune activity, suggesting the tremendous clinical potency of this molecular subtype. As is elucidated above, the transcription profile of IRLs is tightly correlated with immune cells infiltration, tumor purities, and immune status. In short, they are closely connected with the immune landscape of UCEC. Accumulating evidence suggests a crucial role of TME in assessing prognosis of several tumors (49). Therefore, we came up with the idea to use IRLs as a risk signature to forecast the clinical outcome of UCEC patients.

The full profile of IRLs transcription is not a practical tool for clinical use, due to the availability of full transcriptome sequencing. This process also generates excessive data, which is almost impossible for care providers to analyze in clinical settings. Consequently, we performed the LASSO regression to set up an IRLs-based signature which consisted of five key IRLs. Moreover, our proposed IRLPS showed a superior precision to its predecessors (37, 38). To achieve better performance of IRLPS, we further constructed a nomogram by integrating risk score and other clinical factors. Calibration curves showed the nomogram had favorable ability for survival assessment. Next, GSEA analysis indicated that ERBB signaling, TGF-β signaling, and Wnt signaling were enriched in the high-risk group, suggesting patients with high risk tend to have a pro-tumor effect. It is believed that the intracellular accumulation of β-catenin is a marker for the activation of the classical Wnt signaling pathway, so any mutation genes resulting in the accumulation of β-catenin will activate the classical Wnt signaling pathway. Wnt signaling pathway, one of the main factors inducing the occurrence of cancer metastasis, could upregulate the expression of Slug, Snail, and Twist and block the expression of E-cadherin, causing the lack of epithelial polarity and connection (50). Almost 40% of UCEC cases exhibit abnormal activation of the Wnt/β-catenin pathway. It has been shown that CT-NNB1 mutations leading to activation of the Wnt signaling pathway are bound up with high-grade UCEC in young women (51). As suggested by Chen et al., inhibition of MRP4 could block the viability and survival of endometrial tumors by targeting Wnt/β-catenin pathway (52).

In our established IRLPS, five model IRLs (HMGN3-AS1, LEMD1-AS1, AP000880.1, AC244517.1, and AC011466.1) were deeply involved in the pathological processes of UCEC. HMGN3 is involved in glucose transportation in cells (53), DNA binding, protein binding (54), and chromatin organization (55). LEMD is found to promote proliferation in gastric cancer *via* activating the PI3K/Akt signaling pathway (56), and is also found to be active in tumorigenesis in colorectal cancer (57) and prostate cancer (58). AP000880.1 is possibly related to TTC12 and NCAM1 gene, which in turn plays an important role in the initiation of leukemia (59, 60). AC244517.1 is associated with the PCDHB family gene, which regulates protocadherin, and is responsible for cell-to-cell adhesion (55) and synaptic transmission (61). AC011466.1 is associated with ZSWIM9, CARD8, PLA2G4C, and LIG1 gene. In research by Linder et al. in 2020, CARD8 can promote T cell proptosis *via* the CARD8-caspase-1-GSDMD axis (62). LIG1 has already seen comprehensive research, and its role in DNA ligase activity (63) and DNA repair (64, 65) is well established. Li et al. reported in a meta-analysis that included 10 studies with a total of 4012 lung cancer cases and 5629 healthy controls that upregulated expression of LIG1 is related to the increased risk of lung cancer (66). However, due to the limited clinical samples, the results of the PCR were not completely consistent with the bioinformatics analysis. Tumor is a complicated disease induced by multigene, since the interaction of genes contribute to the complexity of tumor regulatory mechanisms.

As a current research hotspot, immune activity plays a central part in tumor development. Our model can successfully demonstrate the capability of mirroring immune status and evaluating the benefits of immunotherapy. By depicting the immune landscape of two risk groups, we observed that risk score exhibited a negative correlation with immunescore which is an indicator of immune activity in TME, suggesting high-risk patients were prone to an immunosuppressive status. CIBERSORT disclosed that M2 macrophages were greatly enriched in the high-risk group. As a type of immunosuppressive immunocyte, M2 macrophages have been proven to be closely bound up with poor patient outcome of UCEC, which is in agreement with the results predicted by our IRLPS.

ICI is currently an effective treatment which could strengthen immune activity of the human body by blocking immune escape of tumors. We found that four classical immune checkpoints were lowly expressed in the IRLPS-high group, suggesting patients may hardly benefit from ICI therapy. Also, four IPS-related scores were lower in the high-risk group, indicating unsatisfactory immune efficacy of UCEC. TMB is another favorable indicator for evaluating outcomes of immunotherapy and high TMB tends to forecast a poor prognosis. We demonstrated that TMB value was significantly higher in the IRLPS-high group. All the above results suggest that our model can predict the benefit of immunotherapy for UCEC patients and offer a valuable reference for individualized treatment.

In addition to immunotherapy, we sought to determine the association between risk score and the effectiveness of common chemotherapeutic agents in managing UCEC. We found that the high-risk group had lower IC50 of etoposide and doxorubicin. This means that patients with high IRLPS might benefit from these two drugs. Apart from the conventional drugs, we also explored several promising small molecule agents such as imiquimod, fluphenazine, and cabozantinib which can interact with model IRLs. Imiquimod is an aminoquinoline immune modulator that induces interferon production and activates innate immune cells *via* TLR-7, and thus initiates apoptotic and autophagic cell death (67–69). Fluphenazine is a potent antipsychotic drug, dating back to its discovery in the 1950s, exerting its effect by blocking dopamine receptors (70). Cabozantinib is a tyrosine kinase inhibitor, known for inhibiting VEGFR, MET, and AXL, already in clinical use against multiple kinds of malignancies like hepatocellular carcinoma (71), sarcoma (72), and renal-cell carcinoma (73).

This research still has several limitations. First, the clinical and expression data we used for our research are mainly TCGA-based, and thus limited in sample size, patients race, and ethnicity, which should be validated in larger and localized sets of examples. Second, our analysis is based on our choosing of the algorithm, and although we spared no effort in tuning and optimization, there will still be a certain amount of bias in our model. Third, the link we observed between IRL transcription and TME is correlational, not causal. Further investigation *in vivo* is needed to confirm the interaction of IRLs with other components of TME.

## Conclusion

In this study, we identified a novel inflammation-related subtype of UCEC. On the basis of five hub prognostic IRLs (HMGN3-AS1, LEMD1-AS1, AP000880.1, AC244517.1, and AC011466.1), a robust risk signature was created which could serve as an independent clinical factor for UCEC. Our nominated signature cannot only mirror the immune landscape and assess immunotherapy response for UCEC cases, but also provide valuable chemotherapeutic strategies for individualized treatment.

## Data Availability Statement

The original contributions presented in the study are included in the article/**Supplementary Material**. Further inquiries can be directed to the corresponding authors.

## Author Contributions

HZ and XT visualized the study and took part in the study design and performance. HG, JS, YC, and YW conducted the manuscript writing and bioinformatics analysis. All authors read and approved the final manuscript.

## Conflict of Interest

The authors declare that the research was conducted in the absence of any commercial or financial relationships that could be construed as a potential conflict of interest.

## Publisher’s Note

All claims expressed in this article are solely those of the authors and do not necessarily represent those of their affiliated organizations, or those of the publisher, the editors and the reviewers. Any product that may be evaluated in this article, or claim that may be made by its manufacturer, is not guaranteed or endorsed by the publisher.

## Supplementary Material

Supplementary Figure 1 Class discovery *via* consensus clustering to assigned patients into two clusters. **(A)** Consensus index of clustering models with CDF for k = 2–9 (k means cluster count). **(B)** Consensus clustering matrix for k = 2. **(C)** Consensus clustering model with CDF for k = 2–9. **(D)** Relative change in area under the CDF curve for k = 2–9.

Supplementary Figure 2 Multiple GSEA analysis was used to predict the potential functions and pathways involved in the clusters. **(A)** The enriched KEGG pathways involved in cluster 1. **(B)** The enriched KEGG pathways involved in cluster 2.

Supplementary Figure 3 The coefficients of the 27 IRL signatures evaluated by multivariate Cox regression with LASSO.

Supplementary Figure 4 Transcription of five IRLs involved in our risk signature. **(A–E)** The transcription level of **(A)** LEMD1-AS1, **(B)** HMGN3-AS1, **(C)** AP000880.1, **(D)** AC244517.1, and **(E)** AC011466.1, differs significantly between the groups. **(F–J)** RT-qPCR were conducted to validate this difference in clinical samples. The transcription level of IRLs **(G)** LEMD1-AS1 and (H) AP000880.1 differs significantly between normal and tumor samples.

Supplementary Figure 5 PCA and alluvial plot are utilized to explore the distribution of patients into risk groups. **(A–C)** Principal components analysis between the groups in **(A)** training set, **(B)** testing set, and **(C)** the entire set. **(D)** Alluvial diagram of patients in risk-stratified groups distributed in different gene cluster and survival outcomes.

Supplementary Figure 6 The transcription level of **(A)** M6A methylation related genes and **(B–D)** Mismatch repair genes in high-risk and low-risk group.

## Abbreviations

UCEC, Uterine corpus endometrial carcinoma; TCGA, The cancer genome atlas; IRLs, inflammation-related lncRNA; TMB, tumor mutation burden; ICIs, immune checkpoint inhibitors; GO, Gene ontology; KEGG, Kyoto encyclopedia of genes and genomes; GSEA, Gene set enrichment analysis; OS, Overall survival; IRLPS, IRLs prognostic signature.
