# Supplementary figures and images for: Inflammation-Related LncRNAs Signature for Prognosis and Immune Response Evaluation in Uterine Corpus Endometrial Carcinoma
Source: Front Oncol. 2022 Jun 2;12:923641. doi: 10.3389/fonc.2022.923641 (PMC9201290; doi:10.3389/fonc.2022.923641)

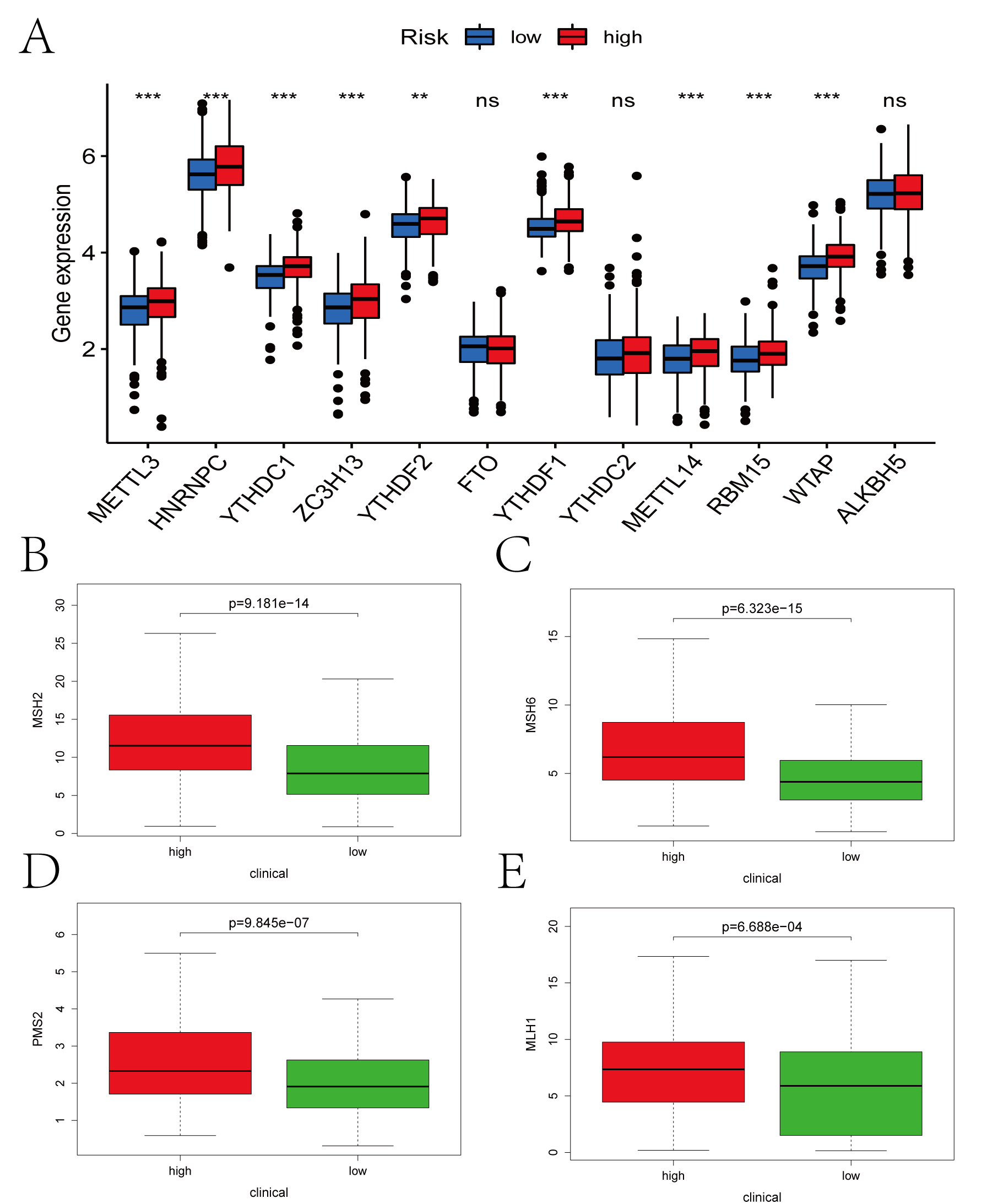

Supplement: Supplementary Figure 1 — Class discovery via consensus clustering to assigned patients into two clusters. (A) Consensus index of clustering models with CDF for k = 2–9 (k means cluster count). (B) Consensus clustering matrix for k = 2. (C) Consensus clustering model with CDF for k = 2–9. (D) Relative change in area under the CDF curve for k = 2–9. [file Image_1.tif]

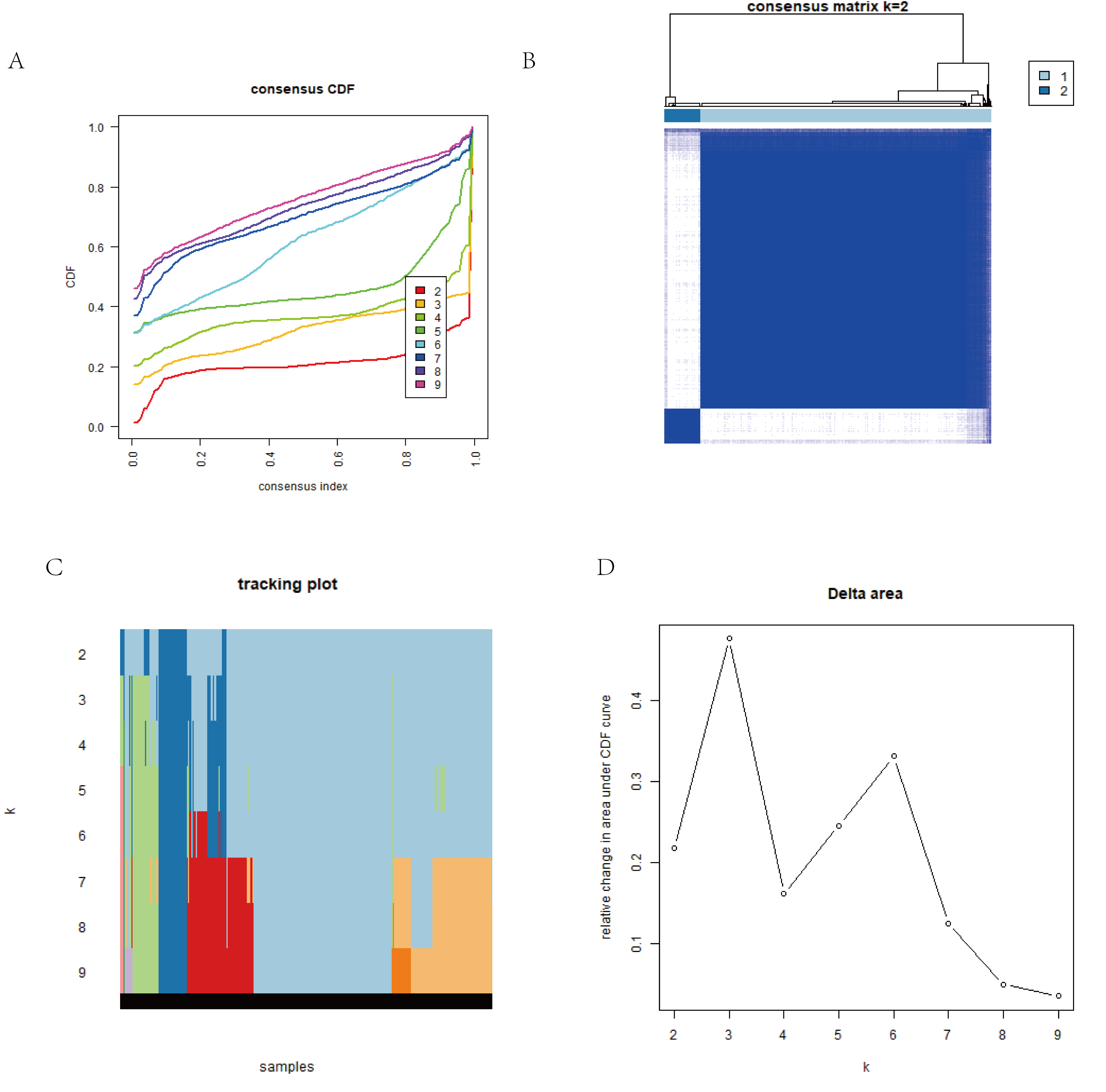

Supplement: Supplementary Figure 2 — Multiple GSEA analysis was used to predict the potential functions and pathways involved in the clusters. (A) The enriched KEGG pathways involved in cluster 1. (B) The enriched KEGG pathways involved in cluster 2. [file Image_2.tif]

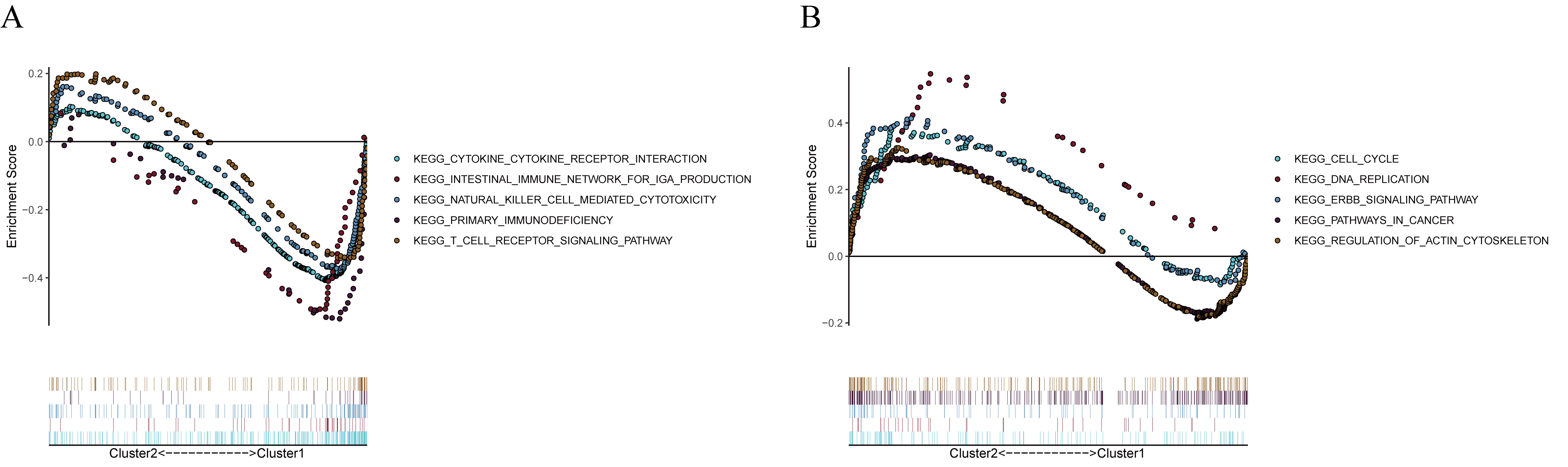

Supplement: Supplementary Figure 3 — The coefficients of the 27 IRL signatures evaluated by multivariate Cox regression with LASSO. [file Image_3.tif]

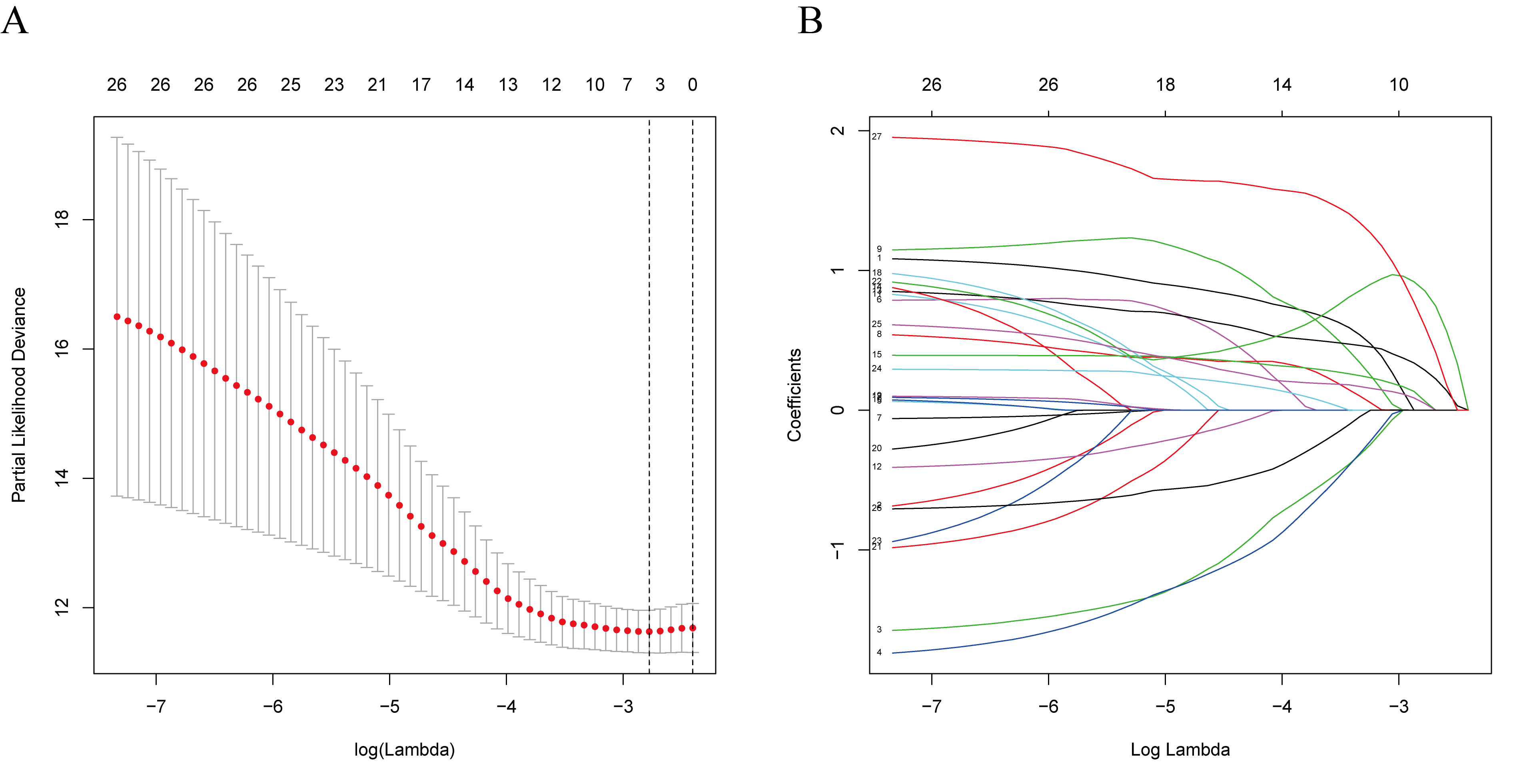

Supplement: Supplementary Figure 4 — Transcription of five IRLs involved in our risk signature. (A–E) The transcription level of (A) LEMD1-AS1, (B) HMGN3-AS1, (C) AP000880.1, (D) AC244517.1, and (E) AC011466.1, differs significantly between the groups. (F–J) RT-qPCR were conducted to validate this difference in clinical samples. The transcription level of IRLs (G) LEMD1-AS1 and (H) AP000880.1 differs significantly between normal and tumor samples. [file Image_4.tif]

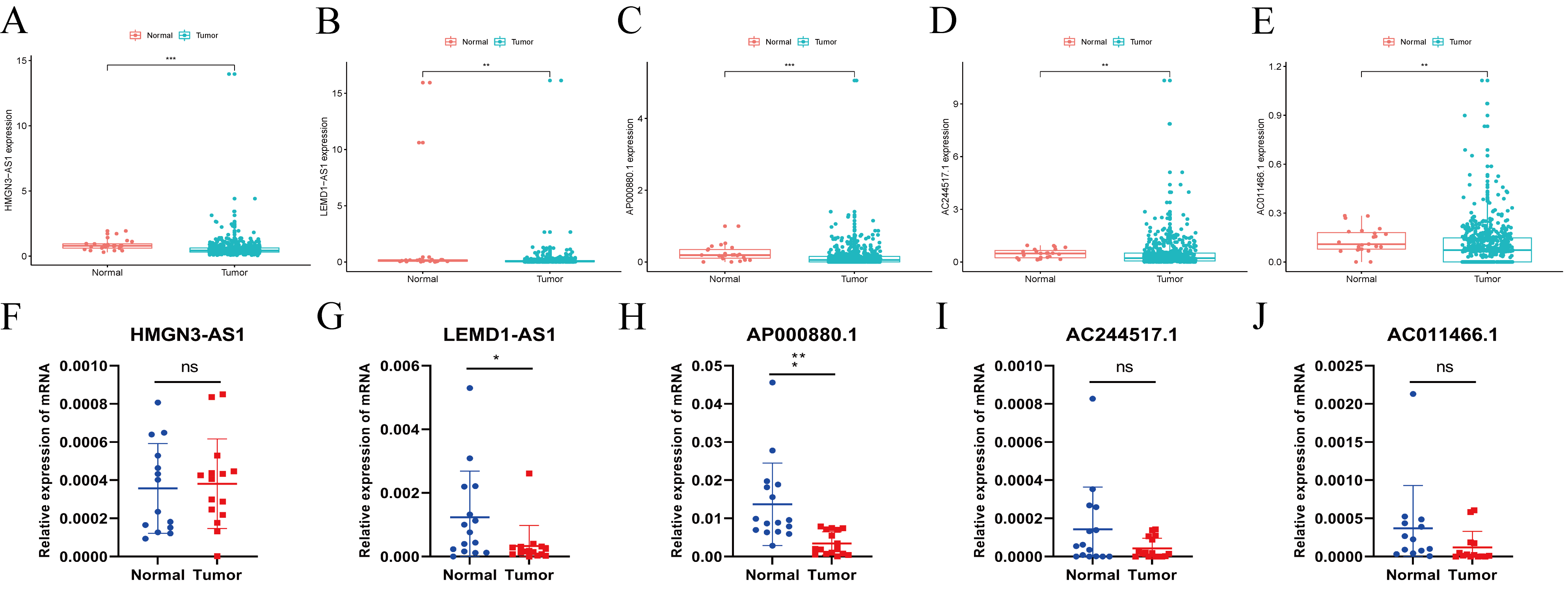

Supplement: Supplementary Figure 5 — PCA and alluvial plot are utilized to explore the distribution of patients into risk groups. (A–C) Principal components analysis between the groups in (A) training set, (B) testing set, and (C) the entire set. (D) Alluvial diagram of patients in risk-stratified groups distributed in different gene cluster and survival outcomes. [file Image_5.tif]

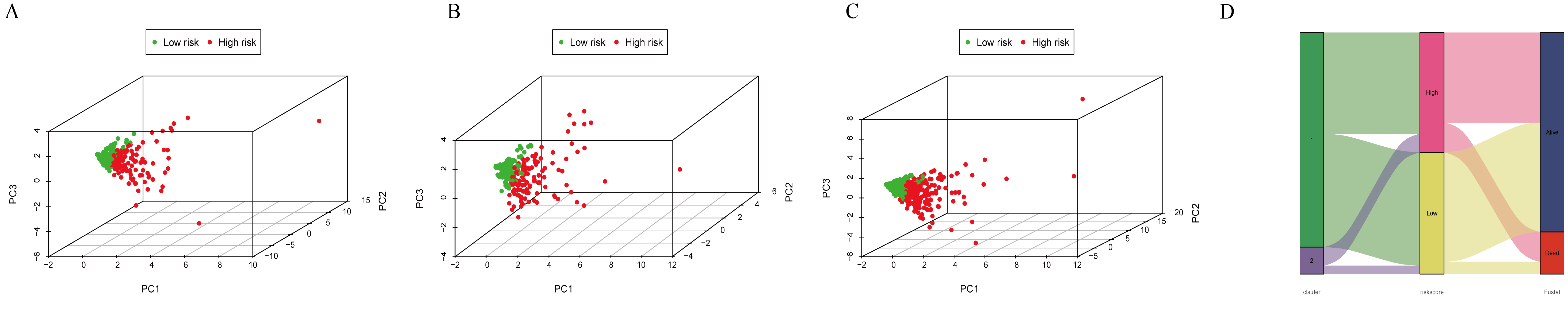

Supplement: Supplementary Figure 6 — The transcription level of (A) M6A methylation related genes and (B–D) Mismatch repair genes in high-risk and low-risk group. [file Image_6.tif]
