# Supplementary material for: Inflammation-Related LncRNAs Signature for Prognosis and Immune Response Evaluation in Uterine Corpus Endometrial Carcinoma
Source: Front Oncol. 2022 Jun 2;12:923641. doi: 10.3389/fonc.2022.923641 (PMC9201290; doi:10.3389/fonc.2022.923641)
Supplement: Supplementary file 7 [file Table_1.docx]

| **Table S1. Distribution of patients into training cohort, testing cohort and total cohort.** | | | | | |
| --- | --- | --- | --- | --- | --- |
| Covariates | Type | Total | Train | Test | Pvalue |
| age | <=60 | 199(38.94%) | 111(43.36%) | 88(34.51%) | 0.05 |
|  | >60 | 312(61.06%) | 145(56.64%) | 167(65.49%) |  |
| histological type | endometrial | 384(75.15%) | 192(75%) | 192(75.29%) | 1 |
|  | Mixed and serous | 127(24.85%) | 64(25%) | 63(24.71%) |  |
| grade | G1 & G2 | 91(17.81%) | 48(18.75%) | 43(16.86%) | 0.66 |
|  | G3 & G4 | 420(82.19%) | 208(81.25%) | 212(83.14%) |  |
| stage | Stage I & Stage II | 370(72.41%) | 183(71.48%) | 187(73.33%) | 0.71 |
|  | Stage III Stage IV | 141(27.59%) | 73(28.52%) | 68(26.67%) |  |
