# Supplementary material for: Inflammation-Related LncRNAs Signature for Prognosis and Immune Response Evaluation in Uterine Corpus Endometrial Carcinoma
Source: Front Oncol. 2022 Jun 2;12:923641. doi: 10.3389/fonc.2022.923641 (PMC9201290; doi:10.3389/fonc.2022.923641)
Supplement: Supplementary file 8 [file Table_2.docx]

**Table S2. Primers used in qPCR process to determine transcription level of prognostic IRLs.**

| IRLs | Forward primers | Reverse primers |
| --- | --- | --- |
| HMGN3-AS1 | AAATCTGCCTGGCGACTGA | GAGAGAGCAGCGAAACAATGA |
| LEMD1-AS1 | AAGAGGAGAGTAATGACCGCAA | CCGTGATGAACACAAATGCC |
| AP000880.4 | GGTCAGCACAGCCCTTTAGA | ACAAGTCTTTACCCGTGAGTCT |
| AC244517.2 | CGGAATCCGCTAAGACCGAG | TGGGCAGTCTACTGGACTCA |
| AC011466.6 | CAGGCTCACCTCTTCAGTGC | CTCCTGCGAGGTGTAGAACC |
