# Supplementary material for: Inflammation-Related LncRNAs Signature for Prognosis and Immune Response Evaluation in Uterine Corpus Endometrial Carcinoma
Source: Front Oncol. 2022 Jun 2;12:923641. doi: 10.3389/fonc.2022.923641 (PMC9201290; doi:10.3389/fonc.2022.923641)
Supplement: Supplementary file 9 [file Table_3.docx]

| **Table S3. Univariate Cox analysis generated 27 IRLs that are significantly related to the overall survival (OS) of UCEC patients.** | | | | |  |
| --- | --- | --- | --- | --- | --- |
| gene | HR | Lower 95% CI | Higher 95% CI | pvalue | |
| CFAP58-DT | 2.1162 | 1.2593 | 3.5560 | 0.0046 | |
| FAM66C | 3.1960 | 1.5437 | 6.6166 | 0.0018 | |
| UNQ6494 | 0.0081 | 0.0004 | 0.1452 | 0.0011 | |
| AC078883.1 | 0.3806 | 0.1852 | 0.7820 | 0.0086 | |
| AL049539.1 | 1.4328 | 1.1414 | 1.7988 | 0.0019 | |
| AP000593.3 | 3.5878 | 1.5055 | 8.5505 | 0.0039 | |
| AP002761.4 | 1.1009 | 1.0491 | 1.1553 | 0.0001 | |
| AL645568.1 | 3.3838 | 1.8640 | 6.1428 | 0.0001 | |
| NBAT1 | 1.3232 | 1.0718 | 1.6336 | 0.0092 | |
| FMR1-IT1 | 1.4578 | 1.1154 | 1.9054 | 0.0058 | |
| LRRC8C-DT | 3.6737 | 1.4920 | 9.0455 | 0.0047 | |
| AL133243.2 | 1.2408 | 1.0780 | 1.4282 | 0.0026 | |
| HMGN3-AS1 | 1.4796 | 1.1078 | 1.9763 | 0.0080 | |
| TPM1-AS | 2.1302 | 1.3048 | 3.4778 | 0.0025 | |
| LEMD1-AS1 | 1.3807 | 1.2028 | 1.5849 | 0.0000 | |
| AC092436.2 | 1.6329 | 1.1589 | 2.3007 | 0.0051 | |
| SOS1-IT1 | 1.2903 | 1.1360 | 1.4657 | 0.0001 | |
| LINC01126 | 2.5007 | 1.2655 | 4.9414 | 0.0083 | |
| AC092953.2 | 2.2176 | 1.2689 | 3.8758 | 0.0052 | |
| AL031667.3 | 1.7529 | 1.1444 | 2.6849 | 0.0099 | |
| AL035530.2 | 4.4538 | 1.8565 | 10.6848 | 0.0008 | |
| AP000880.1 | 3.8437 | 1.7750 | 8.3231 | 0.0006 | |
| AC114947.2 | 2.8938 | 1.3980 | 5.9898 | 0.0042 | |
| AC244517.7 | 1.8161 | 1.2804 | 2.5758 | 0.0008 | |
| AC244517.1 | 1.4300 | 1.1339 | 1.8034 | 0.0025 | |
| AL606970.1 | 0.1665 | 0.0486 | 0.5707 | 0.0043 | |
| AC011466.1 | 16.8753 | 3.4921 | 81.5499 | 0.0004 | |
