# Supplementary material for: Inflammation-Related LncRNAs Signature for Prognosis and Immune Response Evaluation in Uterine Corpus Endometrial Carcinoma
Source: Front Oncol. 2022 Jun 2;12:923641. doi: 10.3389/fonc.2022.923641 (PMC9201290; doi:10.3389/fonc.2022.923641)
Supplement: Supplementary file 10 [file Table_4.docx]

**Table S4. Univariable and multivariable cox regression analysis of the IRLncSig and overall survival in different patient sets.**

| Variable | Univariable model | | | Multivariable model | | |
| --- | --- | --- | --- | --- | --- | --- |
|  | HR | 95%CI | P-value | HR | 95%CI | P-value |
| training set |  |  |  |  |  |  |
| (n=256) |  |  |  |  |  |  |
| age | 1.6210 | 0.8648-3.0383 | 0.1319 |  |  |  |
| histological type | 2.8247 | 1.5361-5.1944 | 0.0008 | 1.5224 | 0.7615-3.0436 | 0.2344 |
| grade | 3.1205 | 1.1075-8.7923 | 0.0313 | 1.6988 | 0.5599-5.1541 | 0.3494 |
| stage | 2.9693 | 1.6280-5.4158 | 0.0004 | 1.8144 | 0.9084-3.6239 | 0.0914 |
| riskScore | 2.6176 | 1.9401-3.5316 | 0.0000 | 2.1336 | 1.5341-2.9673 | 0.0000 |
| testing set |  |  |  |  |  |  |
| (n=255) |  |  |  |  |  |  |
| age | 2.0045 | 0.9648-4.1646 | 0.0623 |  |  |  |
| histological type | 3.2802 | 1.8368-5.8578 | 0.0001 | 2.2620 | 1.2302-4.1593 | 0.0086 |
| grade | 3.7605 | 0.9111-15.5207 | 0.0671 |  |  |  |
| stage | 5.9548 | 3.2695-10.8455 | 0.0000 | 5.1935 | 2.8013-9.6284 | 0.0000 |
| riskScore | 1.3306 | 1.0468-1.6915 | 0.0196 | 1.2776 | 0.9058-1.8021 | 0.1627 |
| all patients |  |  |  |  |  |  |
| (n=511) |  |  |  |  |  |  |
| age | 1.7782 | 1.1121-2.8432 | 0.0162 | 1.5932 | 0.9780-2.5953 | 0.0614 |
| histological type | 3.0435 | 2.0032-4.6242 | 0.0000 | 1.6275 | 1.0254-2.5831 | 0.0388 |
| grade | 3.3631 | 1.4671-7.7097 | 0.0042 | 1.4165 | 0.5827-3.4432 | 0.4423 |
| stage | 4.1162 | 2.7000-6.2754 | 0.0000 | 3.3973 | 2.1380-5.3981 | 0.0000 |
| riskScore | 1.5440 | 1.3177-1.8092 | 0.0000 | 1.4896 | 1.2215-1.8165 | 0.0001 |
